# NiB-CrC Coatings Prepared by Magnetron Sputtering Using Composite Ceramic NiCr-BC Target Produced by Detonation Spray Coating

**DOI:** 10.3390/nano12203584

**Published:** 2022-10-13

**Authors:** Viacheslav Sirota, Sergei Zaitsev, Dmitriy Prokhorenkov, Mihail Limarenko, Andrey Skiba, Marina Kovaleva

**Affiliations:** 1 Centre for High Technologies, Belgorod State Technological University Named after V.G. Shoukhov, Kostyukov 46, 308012 Belgorod, Russia; 2 Joint Research Center «Technology and Materials», Belgorod State National Research University, Pobeda 85, 308015 Belgorod, Russia

**Keywords:** borides, films, microstructure, magnetron sputtering, multi-chamber detonation device

## Abstract

A metal–ceramic composite target for magnetron sputtering was fabricated for the first time by a robotic complex for the detonation spraying of coatings equipped with a multi-chamber detonation accelerator. A mixture of metal and ceramic NiCr/B_4_C powders was sprayed onto the copper base of the cylindrical composite target cathode. The study of the structure of a metal–ceramic composite coating target using scanning electron microscopy showed that the coating material is dense without visible pores; the elemental composition is evenly distributed in the material. The study of the cathode sputtering area after deposition in the DC mode showed that there are uniform traces of annular erosion on the target surface. The obtained cathode target with an NiCr-70B_4_C coating was used to deposit the NiB-Cr_7_C_3_ coating on flat specimens of 65G steel using equipment for magnetron sputtering UNICOAT 200. The coating was applied in the Direct Current mode. A dense NiB-Cr_7_C_3_ coating with a thickness of 2 μm was obtained. The NiB-Cr_7_C_3_ coating has a quasi-amorphous structure. The microstructures and concentration of oxygen and carbon impurities throughout the entire thickness of the coating were investigated by means of transmission electron microscopy. The results of the study show that the coatings have a nanocrystalline multi-phase structure. The microhardness of the NiB-Cr_7_C_3_ coating reached 10 GPa, and the adhesion fracture load exceeded 16 N. The results will open up new prospects for the further elaboration of technology for obtaining original composite cathodes for magnetron sputtering using detonation spraying of coatings.

## 1. Introduction

Due to the development of the aerospace, automotive, chemical and electrical industries, the requirements for the properties of functional and protective coatings are increasing. Studies show that multi-component coatings obtained by synthesis from a combination of materials with different elemental compositions have unique functional properties [1]. To create high-temperature coatings, it is advisable to use borides and carbides of refractory metals, which have a high melting point and chemical resistance, excellent hardness, and thermal and electrical conductivity of a metallic nature [2,3,4,5,6,7,8,9]. The main methods of obtaining such coatings are the methods of magnetron sputtering of mosaic multi-component cathodes, or simultaneous sputtering of several cathodes simultaneously.

The high hardness and brittleness of the raw materials used for the manufacture of multi-component targets limits the possibilities of using casting technologies, hot pressure treatment, cutting, etc. In these cases, powder technologies are usually used. It is possible to use, for the manufacture of targets, self-propagating high-temperature synthesis (SHS) in powder mixtures, in combination with the pressing of a hot porous product [10,11,12,13,14]. The SHS technology forms large internal stresses of the first kind in the product, which arise during hot pressing and subsequent cooling, which limits its use for the manufacture of targets. The SHS method is limited in its elemental composition when creating complex compositions since the SHS reaction is possible only in mixtures with sufficient thermal release [15]. The limitations inherent in the SHS method exclude the possibility of obtaining cylindrical cathodes from ceramics or cermets. In addition, due to the presence of defects on the surface of the sintered powder cathode in the normal operation of the cathode-arc plasma source with a filter, problems arise with the stability of the discharge. The creation of mosaic cathodes with ceramic inserts is extremely laborious to manufacture in comparison with the proposed technology. The known approaches do not make it possible to obtain cylindrical cathodes from ceramics or cermet.

In this article, the possibility of manufacturing a target for the physical deposition of coatings from the vapor phase (magnetron sputtering) using the detonation spray coating (DSC) process [16,17,18] was investigated. To fulfill these tasks, a robotic complex for detonation spraying of coatings (IntelMashin LLC, Moscow, Russia) equipped with a multi-chamber detonation accelerator (MCDS) was developed [18].

The scope of application of detonation technologies is limited mainly by the creation of protective coatings on the surface of quickly worn products. The authors did not find any publications on the use of gas–thermal detonation technologies in the field of manufacturing metal–ceramic composite targets for magnetron sputtering. In connection with the above, the purpose of this work is to show the effectiveness of detonation technology and related devices for obtaining original composite cylindrical cathodes for magnetron sputtering with a ceramic or cermet surface layers, using the example of NiCr + B_4_C powder composition. These materials provide an opportunity to create composite coatings with a unique composition and characteristics using a magnetron.

## 2. Materials and Methods

### 2.1. Powder Preparation

**Figure 1:**
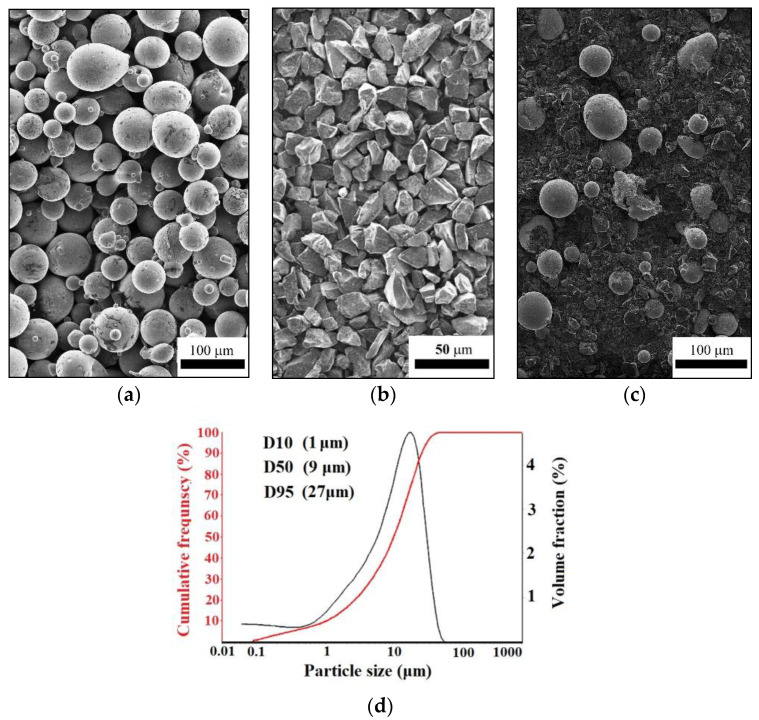
Morphology and particle size of the initial powders and their compositions. Powder morphologies: AP-NiCr17Si4B4 (**a**), B_4_C (**b**) and their composition after mixing (**c**). Particle size distribution of NiCr-70B_4_C powder composition (**d**).

To implement the detonation coating technology, a powder composition was used: AP-NiCr17Si4B4 powder (POLEMA JSC, Tula, Russia) (70Ni–17Cr, impurities 4.3Fe-4.1Si-3.6B-1.0C, all in wt pct) and B4C F600 FEPA powder (Volzhsky Abrasive Plant JSC, Russia) (78B-20C, impurities 0.2B_2_O_3_-0.2Si-0.2Fe-1.0N-0.2C free). The morphology of the initial powders, according to scanning electron microscopy (SEM, TESCAN MIRA 3 LMU, Brno-Kohoutovice, Czech Republic), is shown in Figure 1a,b.

The initial powders were mixed in a ratio of 30 wt% of NiCr and 70 wt% of B_4_C (denoted as NiCr-70B_4_C) in the Turbula mixer for 1 h (Figure 1c). The particle size distribution was measured by the laser scattering method using a particle size analyzer (Analysette 22 NanoTec Plus, Fritsch GmbH, Idar-Oberstein, Germany) (Figure 1d). The powder composition was dried in an electric oven at 200 ± 5 °C for 60 min to reduce agglomeration and eliminate the possibility of sticking during the detonation spray coating process.

### 2.2. Metal–Ceramic Composite Target Preparation

Copper cathode target of equipment for magnetron sputtering UNICOAT 200 (NPF “Elan-praktik”, Dzerzhinsk, Russia) in the form of the rotating cylindrical target (100 mm × 5 mm, Ø 71 mm) was made. A powder NiCr-70B_4_C was sprayed on the surface of cathode targets by a robotic complex for detonation spraying of coatings (IntelMashin LLC, Moscow, Russia) equipped with a multi-chamber detonation accelerator (MCDS) [16,17,18]. The robotic complex was manufactured by IntelMashin LLC (Russia) for Belgorod State Technological University V.G. Shoukhov (Belgorod, Russia).

MCDS provides uniform mixing of the mixture of micro-powders, dosing and periodic supply of powder doses to the nozzle. At the same time, the uniform distribution of micro-powders along the nozzle cross-section is preserved, their stratification is excluded. The MCDS uses an oxygen–butane-based combustible mixture, which forms moderate temperatures of combustion products (up to 2000 °C), which exclude overheating of micro-powders. The high speed of the combustion products is ensured by their accumulation from the cylindrical and hemispherical chambers and the formation of a high pressure region (up to 35 atm.) before entering the nozzle. A rapid increase in the pressure of the combustion products and a large pressure drop forms shock waves in the nozzle and a rapid outflow of the combustion products (up to 1600 m/s). Preliminary injection of a dose of a mixture of micro-powders into the nozzle ensures their heating and acceleration up to 1000 m/s. Heated micro-powders are pressed at high speed onto the target surface, creating a dense coating with a uniform distribution of chemical elements. This device can use two or more devices for dosing and entering a mixture of micro-powders, which increases its flexibility and makes it possible to create a target with a mosaic coating from any mixture of powders.

The multi-chamber detonation device and cumulative technology are widely used in industry to obtain dense coatings with high adhesive and cohesive properties [18,19,20,21,22,23,24,25]. This technology has a huge potential for the production of metal–ceramic composite targets from a mixture of micro-powders due to the following advantages: the ability to spray almost any powder compositions, high deposition efficiency of the coating up to 90%, the possibility of spraying large areas at a relatively low cost of the process, differentiation in thickness and size of the coating. It is possible to suppress the segregation and oxidation of the coating material by controlling the amount of oxygen in the fuel mixture and the formation of a reducing atmosphere during the coating process.

Before deposition of the coating, the surface of the targets was degreased and sandblasted. The parameters of the NiCr-70B_4_C coating spray are listed in Table 1. The study of the structure and distribution of elements in the coating material was carried out by scanning electron microscopy (SEM, TESCAN MIRA 3 LMU, Brno-Kohoutovice, Czech Republic).

### 2.3. Boride–Carbide Coating Preparation

The obtained cylindrical cathode target with NiCr-70B_4_C coating was used to deposit the NiB-Cr_7_C_3_ coating on flat specimens of 65G steel (Fe-0.65C-0.20Si-0.90Mn-0.04P-0.04S-0.20Cu, all in wt pct) using equipment for magnetron sputtering UNICOAT 200.

Before deposition of the coating, the surfaces of the substrates were degreased and cleaned with argon ions for 10 min at a pressure of 8 × 10^−2^ Pa and a voltage at an ion source of 2.2 kV. In the process of forming a coating on a steel substrate, two targets were used as the sprayed material, a standard carbon target with a purity of 99.99% and a copper target with a metal–ceramic composite coating of NiCr-70B_4_C.

The deposition of the NiB-Cr_7_C_3_ coating was carried out using external carbon target with an excess of carbon to reduce the oxygen content in the coating. The binding of oxygen in CO and its removal during the deposition of the coating minimize the oxygen content in the final coating.

The coating was deposited in the Direct Current mode (DC). Parameters of deposition process are given in Table 2.

To study the phase composition of the NiB-Cr_7_C_3_ coatings using an ARL 9900 series X-ray fluorescence spectrometer (Thermo Fisher Scientific, Basel, Switzerland), coatings were applied on freshly cleaved glass.

The specimens with NiCr-70B4C and NiB-Cr_7_C_3_ coatings were transversally cut, mechanically polished and prepared by standard metallographic methods—sectioning, mounting and polishing—for sample preparation. The sample was prepared by grinding with SiC sandpapers with various specifications (200, 500, 800 and 1000#), followed by polishing with 1-μm diamond slurry according to the procedure recommended by Struers company for ceramic coatings. The specimens were cleaned with distilled water and dried at 100 °C for 3 h.

The coating structures were analyzed by scanning electron microscopy (SEM, TESCAN MIRA 3 LMU, Brno-Kohoutovice, Czech Republic) and transmission electron microscopy (TEM).

Bright-field transmission electron microscopy (BF-TEM) images, high angle annular dark field scanning transmission electron microscopy (HAADF-STEM) images and energy-dispersive X-ray (EDX) spectra and maps were taken on an aberration-corrected Titan Themis Z microscope equipped with a Super-X detection system and operated at 200 kV.

To study the coating structures and concentration of oxygen and nitrogen impurities across the thickness of the coating, a sample of steel 65G with an NiCr-70B_4_C coating 1 μm thick was made. Thin foils for TEM studies were prepared from 3-mm disks, ground to a thickness of about 0.05 mm and electropolished from one side in an electrolyte.

The coating hardness was measured by the method of “instrumental indentation” (ISO 14577-1) using a Dynamic Ultra Micro Hardness Tester Shimadzu DUH-211S at 21.86 mN indenter load. The hardness of the coating was determined on three experimental samples, six tests on each. The maximum depth of penetration of the indenter into the coating was 0.25 μm.

To determine the adhesion/cohesive strength, scratch resistance and the coatings’ destruction mechanism, the REVETEST scratch tester of CSM Instruments with a diamond spherical Rockwell C indenter with a radius of 200 μm was used. In all tests, the load increased linearly from 0.9 to 40 N at a scratch rate of 5.5 mm/min. The length of the scratch was 9 mm. The moment of adhesion or cohesive destruction of the coating was recorded visually after testing (using an inverted optical microscope OLYMPUS GX51 equipped with a digital camera), as well as based on changes in acoustic emission and friction coefficient. The minimum (critical) load L_c_, which led to the destruction of the coating, was determined.

## 3. Results

**Figure 2:**
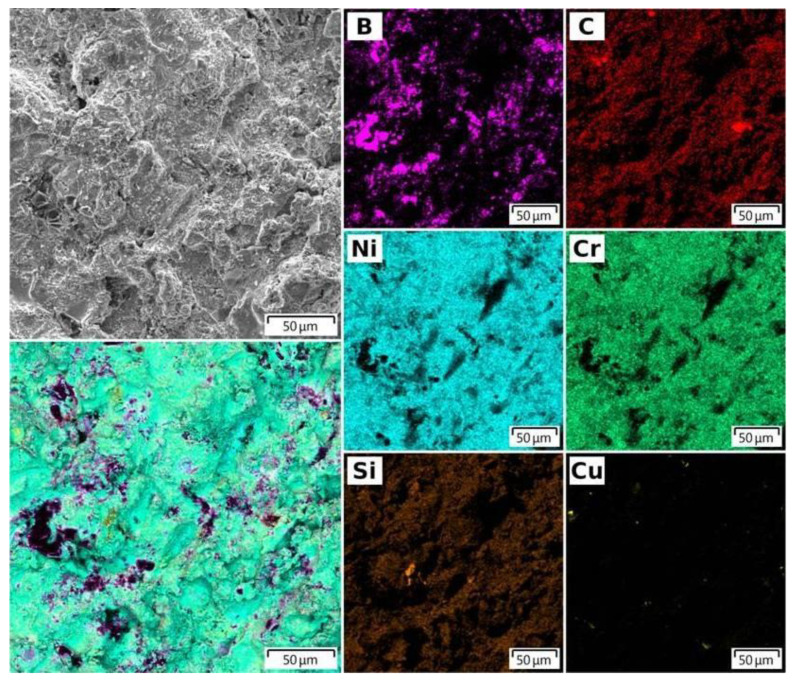
SEM EDX element distribution map of the surface of NiCr-70B_4_C coating on the target surface.

The surface of the 30NiCr-70B_4_C coating of a target for magnetron sputtering was examined by scanning electron microscopy (SEM). It was shown that the surface was obtained as a result of the melting and spreading of NiCr metal powder particles. B_4_C powder particles have a melting point of 2350 °C, which is significantly higher than that of NiCr metal powder (1080 °C). The B_4_C powder particles caused no damage and showed traces of melting and were distributed all over the surface of the coating (Figure 2). The entire surface had a uniform silver-gray color, without visible defects and pores.

**Figure 3:**
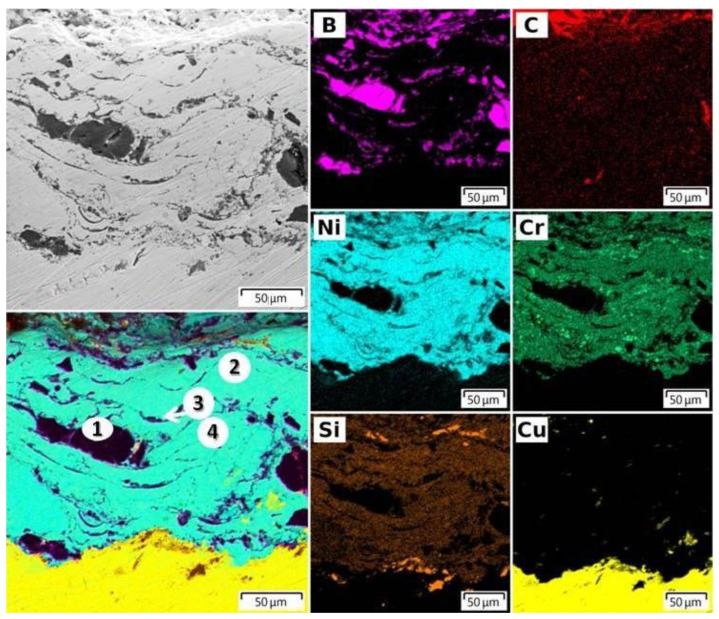
SEM EDX element distribution map of the cross-section of NiCr-70B_4_C coating on the target surface.

The analysis of the distribution of the elements of the mixture of powders (B, C, Ni, Cr, Si) on the surface and in the volume of the NiCr-70B_4_C coating was studied by EDX spectroscopy (Figure 2 and Figure 3). Analysis of the surface and cross-section of the NiCr-70B_4_C coating (Figure 2 and Figure 3) showed the presence of powder mixture elements (B, C, Ni, Cr, Si) both on the surface and in the volume of the coating. This confirms the good mixing of the components of the powder mixture in the process of preparing the mixture and in the process of detonation spraying of the coating. The elemental composition of the cross-sectional surface of a metal–ceramic composite target in accordance with Figure 3 is shown in Table 3.

The coating thickness is 100–150 μm. The coating has a lamella-type structure typical for gas–thermal coatings with inclusions of boron carbide particles.

The target material (Cu) is present in a small amount on the surface of the coating NiCr-70B_4_C. This is due to the fact that copper is a low-melting material, and as a result of the braking of powder particles on the surface of the copper target, it was melted, dispersed and embedded in the coating material.

**Figure 4:**
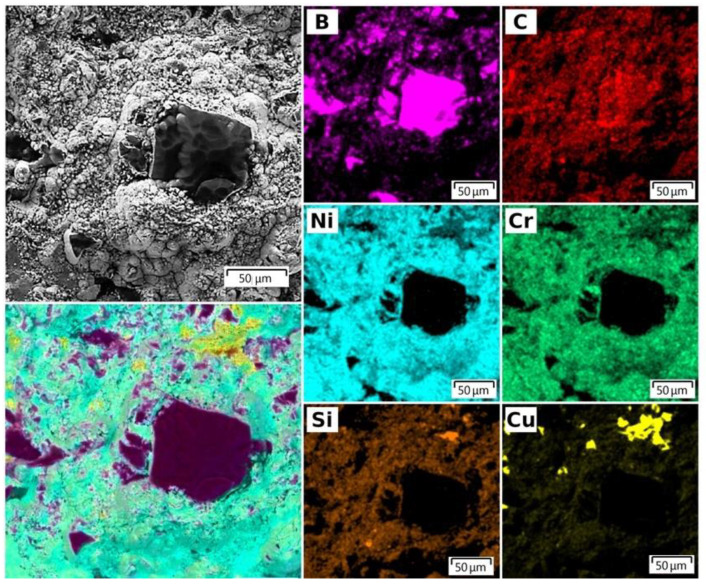
SEM EDX element distribution map of the surface of NiCr-70B_4_C coating on the target surface after deposition of NiB-Cr_7_C_3_ coating.

The SEM image of the erosion area (Figure 4) clearly demonstrates that boron carbide particles are sprayed simultaneously with the metal matrix. EDX spectroscopy analysis of the distribution of the main elements (B, C, Ni, Cr, Si) over the surface of the target coating, after deposition, confirms the uniformity of sputtering of all components.

**Figure 5:**
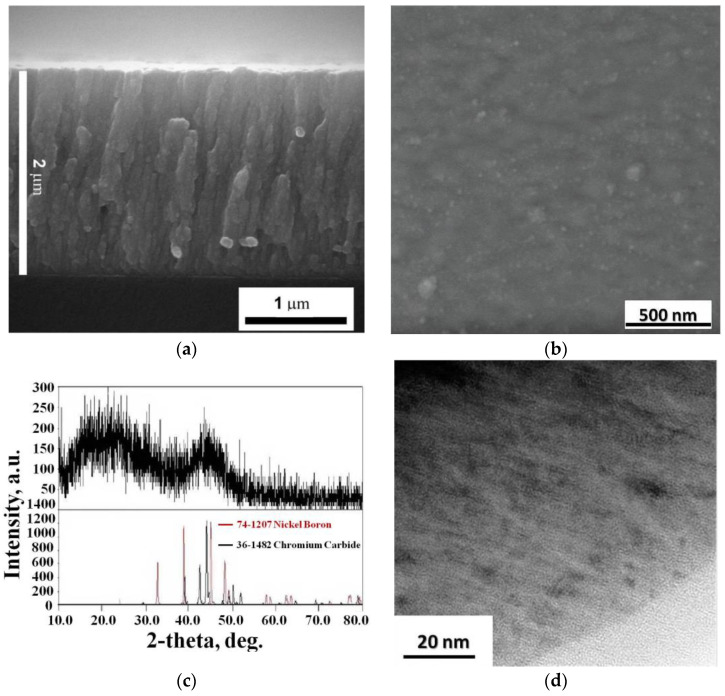
NiB-Cr_7_C_3_ coating on the surface of a 65G steel substrate: SEM image of the fracture (**a**), and the surface (**b**), X-ray phase analysis (**c**), and TEM image (**d**).

In the process of magnetron sputtering, the plasma of the magnetic discharge burns steadily without pulsation. Spraying of a metal–ceramic composite target for 60 min forms traces of annular erosion on the surface of the target. The size of the erosion is uniform over the entire surface of the metal–ceramic composite target. Smooth, dense and continuous NiB-Cr_7_C_3_ coating with a black color with a green tint on the surface of a 65G steel substrate was obtained by magnetron deposition using the obtained target with a coating of NiCr-70B_4_C. NiB-Cr_7_C_3_ coating has a thickness of 2 μm (Figure 5a). The coating material has a nanocrystalline structure, and at the late stages of growth, acquires a textured poly-crystallinity with a size of 100–150 nm (Figure 5b).

The results of studying the structure of the NiB-Cr_7_C_3_ coating (Figure 5d) showed that the coating has a nanocrystalline polyphase structure. The denser phase (dark areas) reaches a size of 10 nm.

The diffractogram (Figure 5c) shows that the NiB-Cr_7_C_3_ coating has a quasi-amorphous structure. The diffraction pattern of the NiB-Cr_7_C_3_ coating shows broad peaks between 10° and 30° and a broader peak around 45°. The presence of broad peaks indicates only short-range ordering or the presence of crystallites smaller than ~2 nm in the coating [26,27]. As shown in Figure 5c, the observed features in the X-ray diffraction patterns can be attributed to different boride (NiB) and carbide (Cr_7_C_3_) phases that can coexist in the deposited coatings. The identification of the phases present in the coating is difficult since several nanocrystalline phases can coexist with a quasi-amorphous matrix, which provides additional peak broadening [28].

The composition of the resulting vacuum coating differs from the material of the cathodes and the composition of the starting ingredients.

The 30NiCr-70B_4_C coating of a target for magnetron sputtering contains up to 15 wt% of oxygen. The deposition of the NiB-Cr_7_C_3_ coating was carried out with an excess of carbon. The binding of oxygen in CO and its removal during the deposition of the coating minimized the oxygen content in the final coating to less than 2 wt% (Table 4, SEM).

The amount of impurities, both metallic and gaseous, is critical for NiB-Cr_7_C_3_ coatings [2,3,4,5,7,8]. In this work, the analysis of carbon and oxygen impurities in the NiB-Cr_7_C_3_ coatings caused by the use of detonation powder spraying technology was carried out.

**Figure 6:**
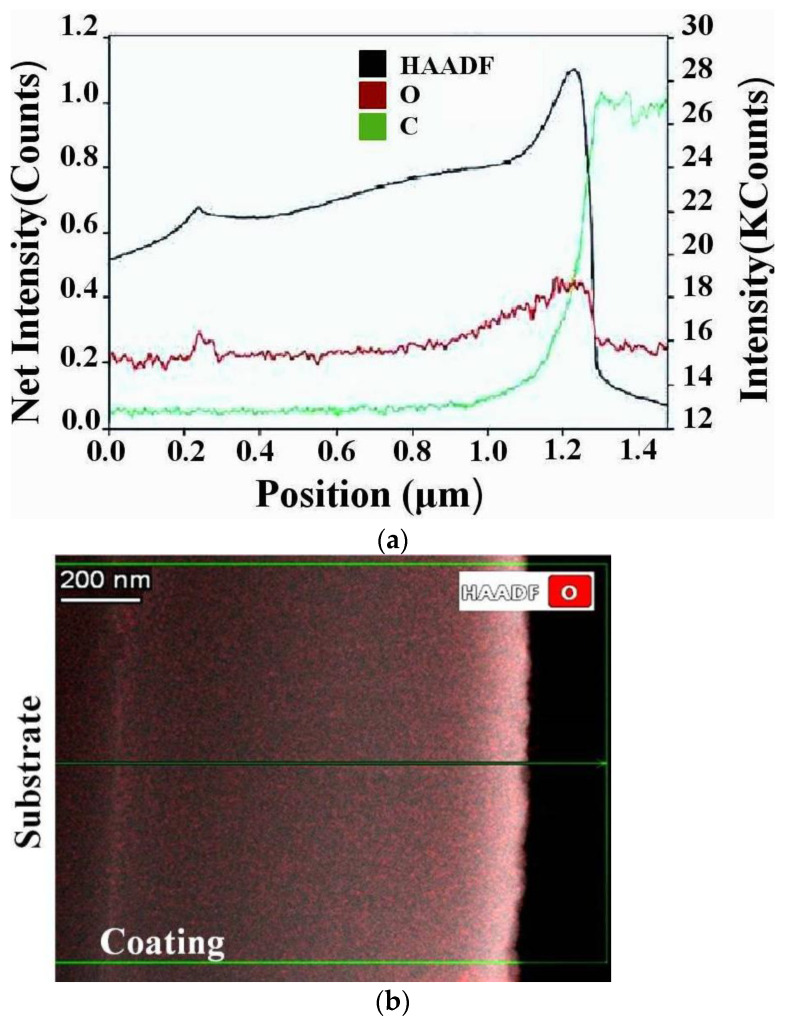
Cross-sectional TEM image of NiB-Cr_7_C_3_ coating (**a**), and EDX spectrum indicating the concentration of oxygen and carbon along red and green lines (**b**).

Figure 6 shows the area of the cross-section of the coating, from which the energy-dispersive analysis of the concentration of oxygen and carbon was carried out using TEM-microscopy methods along the thickness, from the substrate to the surface. The average concentration of oxygen in the coating reaches 5 at. %.

It can be seen that the concentration of oxygen and carbon increases from the “substrate–coating” boundary to the surface of the coating (Figure 6). Two spikes in oxygen levels are also fixed on the spectrum. The first increase in oxygen concentration (coating thickness ~250 nm) is due to the residual presence of oxygen on the surface of the substrate before coating.

The second increase in oxygen concentration (coating thickness ~1150 nm) is partially due to the passivation of the surface of the coating. Residual gases in the vacuum chamber can be sources of additional oxygen atoms also.

Based on the studies carried out, it can be concluded that the resulting vacuum coating is a homogeneous cermet composite consisting of an alloy of metals (NiCr), chromium carbide, nickel boride and an insignificant amount of metal oxides.

**Figure 7:**
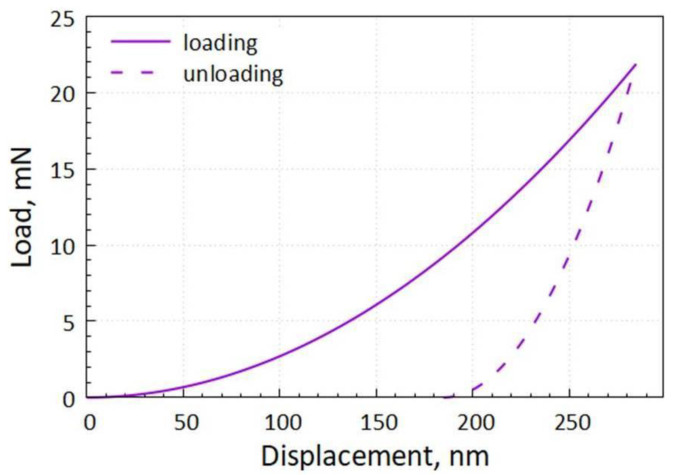
Load–displacement curve of specimen deposited with NiB-Cr_7_C_3_ coating.

The most universal characteristic of the mechanical properties of coatings is microhardness. The microhardness of the coating at an indentation load of 21.86 mN is not less than 10 GPa. Figure 7 shows the load–displacement curve of the specimen deposited with the NiB-Cr_7_C_3_ coating.

**Figure 8:**
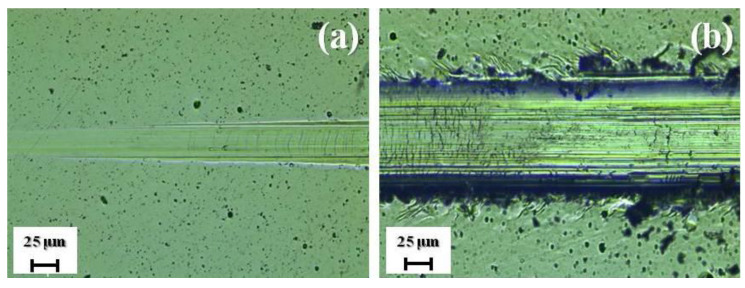
Images of scratch groove formed on the surface of the NiB-Cr_7_C_3_ coating: start point (**a**) and end point (**b**).

The second important characteristic of the mechanical properties of coatings is their adhesive strength. In this paper, for its definition, the method of scratch testing was used. Figure 8 presents the start and end points of the scratch groove formed on the surface of the coating at a progressive load range from 0.9 to 40 N. The coating was not delaminating completely up to a progressive load of 16 N, and the failure of the coating was found in the plastic deformation format. The wear in the coating areas is smooth and with no well-defined cleavages, which is typical for plastic abrasion (Figure 8). When scratched, the coatings become worn out but do not peel off, which is evidence that the cohesion mechanism related to plastic deformation and fatigue facture in the coating material is implemented [29].

## 4. Conclusions

In this study, a robotic complex for detonation spraying of coatings equipped with a multi-chamber detonation accelerator was applied for deposition of the NiCr-70B_4_C coating on the copper surfaces of a cylindrical composite target cathode for magnetron sputtering. The main results can be summarized as follows:

- copper cathode target of equipment for magnetron sputtering in the form of the rotating cylindrical target (100 × 5 mm, Ø 71 mm) was made;
- NiCr-70B_4_C coating (thickness ~100–150 μm) without penetrating cracks was obtained on the surface of the cylindrical target cathode by a detonation sputtering complex;
- NiCr-70B_4_C coating has a lamella-type structure;
- analysis of the surface and cross-section of the NiCr-70B_4_C coating showed the presence of powder mixture elements (B, C, Ni, Cr, Si) both on the surface and in the volume of the coating;
- cylindrical target cathode with NiCr-70B_4_C coating was used to obtain a smooth, dense and continuous NiB-Cr_7_C_3_ coating on the surface of a 65G steel substrate by magnetron deposition;
- NiB-Cr_7_C_3_ coating with a thickness of 2 μm and a quasi-amorphous structure was obtained;
- to study the coating structures and concentration of oxygen and nitrogen impurities across the thickness of the coating, a sample of steel 65G with a NiCr-70B4C coating 1 μm thick was made;
- the average concentration of oxygen in the coating reaches 5 at. %;
- the microhardness of the coating at an indentation depth of 0.25 μm (12.5% of the coating thickness) and at an indentation load of 21.86 mN is not less than 10 GPa;
- the adhesion fracture load exceeds 16 N with plastic and uniform nature of wear.

The methods and technologies presented in this paper for obtaining original composite cylindrical cathodes for magnetron sputtering with ceramic or cermet surface layers make it possible to synthesize a composite nanostructured coating with a predetermined phase composition and characteristics.

## Figures and Tables

**Table 1:** Parameters of NiCr-70B_4_C coating deposition by a robotic complex for detonation spraying of coatings.

Barrel Length, mm	Barrel Diameter, mm	Deposition Distance, mm	Powder Feed Rate, g/h	Flow Rate of Fuel Mixture Components, m^3^/h		
				Oxygen	Propane	Air
500	16	60	700	* 187/ ** 190	* 41/ ** 41	* 94/ ** 82

* Cylindrical form combustion chamber. ** Combustion chamber in the form of a disk.

**Table 2:** Parameters of deposition of the NiB-Cr_7_C_3_ coating on a 65G steel substrate using UNICOAT 200.

Parameters		Meaning
Leaking		0.06 cm^3^/min
Operating pressure		0.17 Pa
Working gas		Ar (99.999% purity)
Total argon flow in the chamber		78 sccm *
Current/Voltage		
Target	NiCr-B_4_C	2 A/506 V
	Carbon	0.8 A/489 V
Frequency		18 kHz
Cathode material		NiCr-B_4_C Carbon (99.999% purity)
Bias		1 A/40 V
Magnetron–sample distance		70 mm
Deposition time		50 min

* sccm—standard cubic centimeters per minute.

**Table 3:** Chemical composition of the cross-section of NiCr-70B_4_C coating on the target surface (Figure 3).

Point	Element Composition, wt%					
	B	C	Ni	Cr	Si	Cu
1	75.49	23.73	0.34	0.08	0.13	-
2	-	7.90	72.96	10.88	4.04	-
3	54.68	20.05	15.14	2.59	0.82	2.64
4	-	8.23	57.53	20.52	2.99	6.30

**Table 4:** Chemical composition of the NiB-Cr_7_C_3_ coating.

Element composition, wt%	B	C	O	Al	Si	Ca	Cr	Fe	Ni
	11.89	8.64	1.09	0.16	3.52	0.22	12.50	3.39	58.60

## Data Availability

The data presented in this study are available on request from the corresponding author.
